# Pituitary hyperplasia secondary to primary hypothyroidism in adolescents: A medical case report and literature review

**DOI:** 10.1002/ccr3.9541

**Published:** 2024-11-05

**Authors:** Xiangfeng Yuan, Jiangyu Zhu, Xiaoyu Su, Huiling Tan, Siqi Wang, Xueying Zheng, Yu Ding, Sumei Li

**Affiliations:** ^1^ Division of Life Sciences and Medicine, Department of Endocrinology, The First Affiliated Hospital of USTC University of Science and Technology of China Hefei Anhui China; ^2^ Department of Endocrinology, Funan Hospital of Traditional Chinese Medicine Fuyang Anhui China; ^3^ Department of Endocrinology, the First Affiliated Hospital of Bengbu Medical College Bengbu Medical College Bengbu Anhui China

**Keywords:** adolescent, case report, pituitary hyperplasia, primary hypothyroidism, review

## Abstract

Prompt and precise diagnosis of pituitary hyperplasia secondary to primary hypothyroidism (PHPH) is crucial to avoid unwarranted pituitary surgery and potential permanent impairment. Although PHPH may present similarly to pituitary adenoma, it is responsive to thyroxine therapy, underscoring the critical role of differential diagnosis in the treatment of adolescent patients.

## 1 INTRODUCTION

Pituitary hyperplasia secondary to primary hypothyroidism (PHPH) represents a relatively uncommon endocrine pathology, with a higher prevalence in adults compared to adolescents.^1^ This condition emerges from a reduction in thyroid hormone (TH) production, which in turn precipitates an increase in thyroid‐stimulating hormone (TSH) levels, leading to the hyperplasia of pituitary TSH cells. Clinically, PHPH can closely resemble a pituitary adenoma in both imaging and laboratory tests. However, these conditions necessitate distinct treatment strategies, with pituitary adenomas typically requiring surgery while PHPH can be managed with thyroxine replacement.^2^ Therefore, distinguishing between these conditions through accurate diagnosis is imperative to prevent unwarranted pituitary surgeries and the potential for irreversible glandular dysfunction. In this article, we explore a pediatric case of PHPH accompanied by pituitary stalk obstruction and delve into the relevant literature concerning PHPH in the adolescent population.

## 2 CASE HISTORY AND EXAMINATION

**FIGURE 1:**
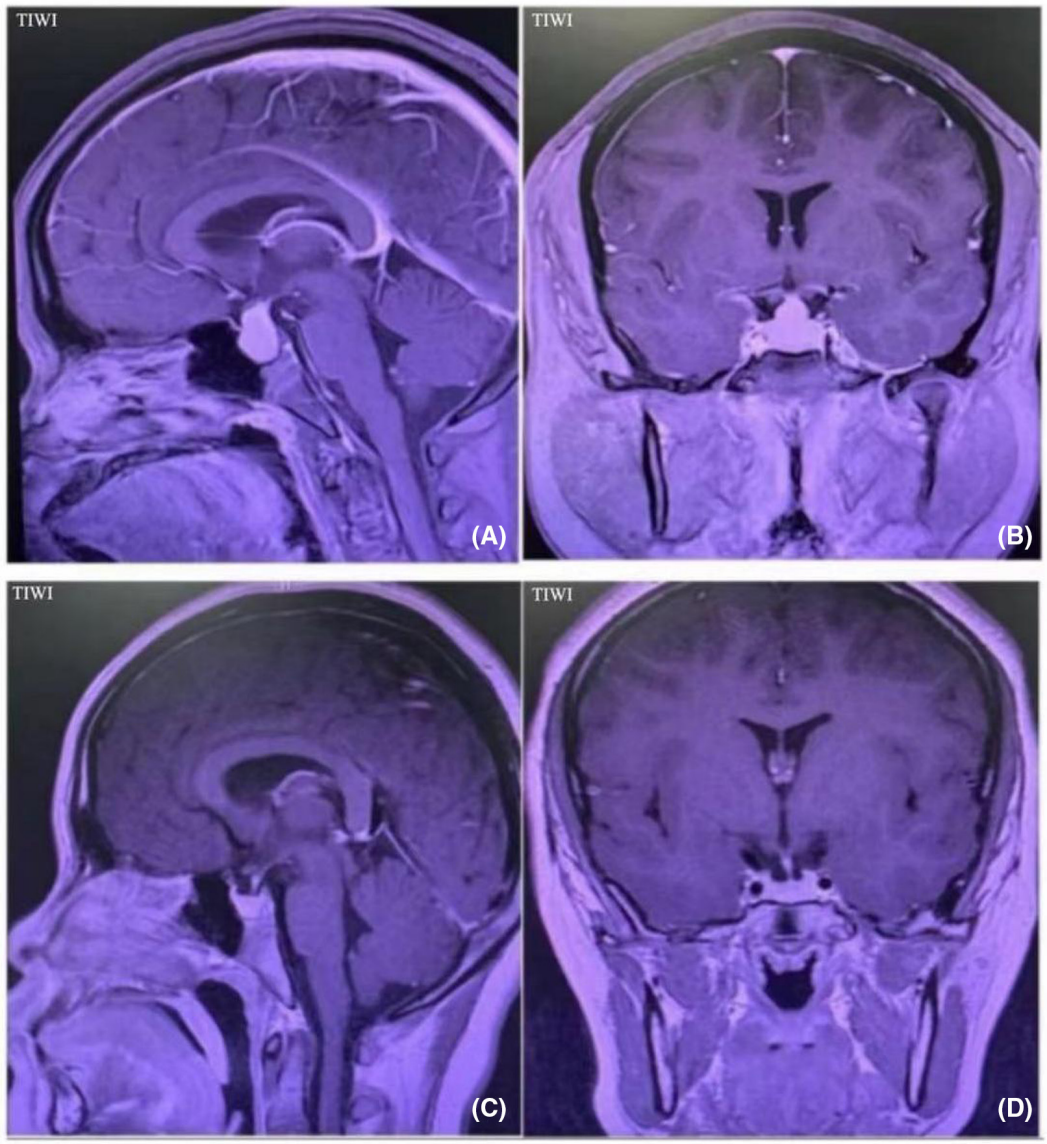
MRI images before and after treatment. (A) MRI enhanced sagittal position of pituitary before treatment; (B) MRI enhanced coronal position of pituitary before treatment; (C) Pituitary MRI enhanced sagittal position after 6 months of treatment; (D) Pituitary MRI enhanced coronal position after 6 months of treatment.

A 13‐year‐old girl began experiencing intermittent headaches 2 years ago without any apparent cause. Although the headaches improved after rest, the child did not seek medical attention at that time and remained undiagnosed and untreated. The child began menstruating 1 year ago, but her menstrual cycle is irregular. Three months ago, the child's family members noticed that she was short and took her to Anhui Children's Hospital. A magnetic resonance imaging (MRI) scan of the sellar area revealed a space‐occupying lesion, raising the possibility of a pituitary tumor. The child has been experiencing intermittent headaches and vision loss for nearly 3 months and for further diagnosis and treatment, she was admitted to Anhui Provincial Hospital's neurosurgery department. An outpatient pituitary magnetic resonance flat scan enhancement revealed a space‐occupying lesion in the pituitary fossa and sella area, measuring approximately 1.1 × 1.7 × 1.8 cm with clear boundaries (Figure 1A,B). The lesion exhibits a “dumbbell‐shaped” and is slightly narrow in the saddle plane, causing compression of the hypophyseal stalk and depression of the saddle bottom. The T1 and T2 signals are enhanced, and the signal is uniform. The optic cross pressure is slightly elevated. The patient has no significant medical history, is unmarried, and has no family history of thyroid disease or autoimmune disease. Physical examination shows a body temperature of 36.8°C, pulse rate of 78 bpm, respiratory rate of 19 bpm, blood pressure of 112/66 mmHg. The patient's height was 139 cm, below the 3rd percentile for age‐matched children (145 cm),^3^ weight was 50 kg, and body mass index (BMI) was 25.88 kg/m.^2^ The visio oculus sinister (VOS) of 0.8 and visio oculus dexter (VOD) of 0.5. Gross visual field measurement showed no significant defects. The patient's thyroid gland was non‐tender and classified as grade II in size. Cardiopulmonary and abdominal examination showed no obvious abnormalities, with no edema in either lower limbs.

## 3 DIAGNOSIS

After admission, the patient underwent further pre‐operative tests and examinations. Laboratory test results and their reference ranges are shown in Table 1. The patient had low levels of free triiodothyronine (FT3) and free thyroxine (FT4). Additionally, there was an elevated level of TSH, along with a significant increase in thyroid peroxide antibody (TPOAb), while thyroid globulin antibody (TGAb) remained within the normal range. The patient's growth hormone (GH) level was 0.3 ng/mL, follicle stimulating hormone (FSH) level was 5.53 IU/L, luteinizing hormone (LH) level was 0.04 IU/L, prolactin level was 59.09 ng/mL, estradiol (E2) level was 55.92 pg/mL, and testosterone (T) level was 0.00 ng/mL. Blood routine, urine routine, and coagulation function tests showed no obvious abnormalities. Biochemical tests revealed elevated levels of aspartate transaminase (AST) (38.6 U/L) and normal levels of alanine aminotransferase (ALT) (19.2 U/L) levels. The patient's potassium level was 3.51 mmol/L, sodium level was 135 mmol/L, chloride level was 102 mmol/L, and albumin level was 41.3 g/L (Note: severe chylous). The cortisol (COR) levels were as follows: COR at 8 am: 108.05 nmol/L, Cortisol at 4 PM: 61.92 nmol/L, Cortisol at 12 PM: 73.39 nmol/L. The results of adrenocorticotropic hormone (ACTH) were as follows: ACTH at 8 AM: 22.10 pg/mL, ACTH at 4 PM: 19.00 pg/mL, ACTH at 12 PM: 21.80 pg/mL. Arc effusion was visible in the bilateral thoracic and pericardial areas on the plain chest CT scan.

**TABLE 1:** Results of follow‐up after thyroid hormone replacement therapy.

	At baseline	Follow‐up duration after thyroid hormone replacement therapy			Reference range
		1 month	4 months	6 months	
Headache	Intermittent	Alleviation	Clear relief	Disappear	–
Vision	Descend	Improve	Significant improvement	Normal	–
Menstrual cycle	Irregular	Resume	Regular	Regular	–
Stature	139	139.5	143	145	145–167.6 cm (3rd‐97rd percentiles of height‐for‐age)
FT4	1.59	13.33	10.66	20.21	10.44–24.88 pmol/L
TSH	>150.0	9.544	25.297	4.879	0.38–4.34 mIU/L
PRL	59.09	9.64	–	–	2.8–29.2 ng/mL
COR (8 AM)	108.05	88	182	224	138–690 nmol/L
ACTH (8 AM)	22.1	13.6	17.7	13.6	7.20–63.40 pg/mL
GH	0.3	–	–	–	0–5 ng/mL
IGF‐1	70.7	–	–	–	170‐527 ng/mL
Na	135	141	–	–	137–147 mmol/L

Abbreviations: ACTH, adrocorticotropic hormone; COR, cortisol; FT4, free thyroxine; GH, growth hormone; IGF‐1, insulin‐like growth factor 1; Na, blood sodium; PRL, prolactin; TSH, thyroid‐stimulating hormone.

Based on the clinical manifestations, imaging findings, and laboratory results, the patient was diagnosed with PHPH, anterior pituitary dysfunction, and pituitary stalk obstruction.

## 4 TREATMENT AND FOLLOW‐UP

Surgery was not considered after communication with the patient's parents, and oral medication was used. The treatment regimen consisted of levothyroxine tablets 75 μg qd po and prednisone 5 mg qd po. One month after treatment, the patient visited an endocrine specialist outpatient clinic and reported the onset of menstrual with improved laboratory values. The treatment regimen was adjusted by discontinuing prednisone and increasing the dose of levothyroxine tablets to 100 μg qd po (Monday–Friday) and 75 μg qd po (Saturday–Sunday). Four months after treatment, the patient revisited the endocrine specialist outpatient clinic and reported regular menstrual cycles with a height of 143 cm. Laboratory tests indicate an increase in FT4 and a decrease in TSH compared to baseline levels. The levothyroxine doses was further adjusted to 125 μg qd po (Monday–Friday) and 100 μg qd po (Saturday–Sunday). Six months after treatment, the patient reported regular menstrual cycles and a height increase to 145 cm. Follow‐up pituitary MRI showed a significant reduction in pituitary mass (Figure 1C,D), with normalization of FT4, PRL and COR levels, and near‐normalization of TSH (as detailed in Table 1).

At the beginning of treatment, the patient presented with low cortisol and serum sodium levels, suggesting the possibility of central adrenal insufficiency secondary to pituitary obstruction. Administering levothyroxine treatment alone in patients with both hypothyroidism and hypocortisolism could pose life‐threatening risks. Therefore, a low dose of prednisone was administered. After a month, blood sodium levels returned to normal, menstruation resumed, and the tumor mass decreased. This led to a reduction in compression on the pituitary gland and restoration of the anterior pituitary function axis. Consequently, exogenous glucocorticoid was discontinued. Following 6 months of thyroid hormone therapy, the patient's hormone levels and clinical symptoms significantly improved, although lifelong height is expected to be lower than ideal due to PHPH.

## 5 DISCUSSION

PHPH is a relatively rare disease in adolescent children, and its diagnosis can be difficult, leading to misdiagnosis or missed diagnosis. However, timely and accurate diagnosis is crucial during puberty, a critical period of growth and development. Proper diagnosis and treatment can help children achieve normal puberty development. We reviewed the literature on PHPH in PubMed and China National Knowledge Infrastructure (CNKI) and analyzed 20 cases of PHPH in puberty. Our analysis encompassed the pathogenesis, clinical manifestations, laboratory tests, imaging findings, differential diagnosis, treatment, and follow‐up of PHPH. The findings are summarized in Appendix A, providing a comprehensive overview of PHPH characteristics during adolescence.

### 5.1 Physiology and pathology

The pathogenesis of PHPH is rooted in thyroxine deficiency, such as that seen in Hashimoto's thyroiditis, which triggers an increase in thyroid‐stimulating hormone‐releasing hormone (TRH) secretion. This, in turn, stimulates the pituitary's thyrotroph cells to release TSH in an attempt to compensate for the low thyroxine levels. However, in primary hypothyroidism, the thyroid gland's failure to produce thyroxine in response to TSH leads to a diminished or absent negative feedback on the hypothalamic–pituitary axis. Consequently, this results in persistent stimulation of the pituitary thyrotroph cells, causing hyperplasia and hypertrophy.^4^ It is essential to differentiate between physiological and pathological pituitary hyperplasia. Physiological hyperplasia typically presents with limited extent, minimal impact on the sellar region, no abnormal hormone secretion, and lacks associated clinical symptoms. It usually resolves spontaneously during specific life stages such as puberty, pregnancy and lactation, and does not necessitate intervention. Conversely, pathological pituitary hyperplasia is characterized by a significant increase in size, a pronounced effect on the sellar space, and is often accompanied by elevated prolactin levels, leading to clinical manifestations like headaches, visual disturbances, galactorrhea, menstrual irregularities, and amenorrhea.

### 5.2 Clinical manifestations

Among the 20 children, there were 7 males and 13 females. Clinical manifestations included growth retardation in 15 cases,^5^, ^6^, ^7^, ^8^, ^9^, ^10^, ^11^, ^12^, ^13^, ^14^, ^15^, ^16^, ^17^ short stature (7 cases of bone age delay, general delay 2–4 years),^5^, ^8^, ^10^, ^11^, ^12^, ^15^, ^16^ weight gain in 8 cases,^5^, ^6^, ^9^, ^12^, ^13^, ^18^, ^19^ headache and rough skin in 7 cases,^5^, ^7^, ^8^, ^9^, ^11^, ^14^, ^15^, ^18^, ^19^, ^20^, ^21^ fatigue and fear of cold in 6 cases,^5^, ^6^, ^7^, ^11^, ^14^, ^15^, ^17^, ^21^, ^22^ vision problems and constipation in 5 cases,^5^, ^7^, ^9^, ^11^, ^12^, ^15^, ^16^, ^18^, ^19^ facial edema in 4 cases,^7^, ^18^, ^20^, ^22^ drowsiness, retardation and hoarseness in 3 cases.^5^, ^6^, ^9^, ^12^, ^18^ Two cases developed menstrual disorders,^18^, ^19^ while hypermenorrhea was observed in two other cases.^7^, ^8^ Other clinical symptoms included anemia, anorexia, memory loss, and pericardial effusion,^9^, ^13^, ^15^, ^20^, ^22^ mucinous edema, acanthosis nigricans, dizziness, insomnia, snoring, wheezing after activity, delayed mobility and dry hair in one case, sparse hair, alopecia, mild hirsutism, pleural effusion, hepatomegaly, ovarian cysts, Hoffman syndrome, and Van Wyk‐Grumbach syndrome (VWGS).^5^, ^8^, ^9^, ^10^, ^11^, ^12^, ^13^, ^18^, ^19^, ^22^

From the above symptoms, we summarized that puberty PHPH, besides PHPH common primary hypothyroidism and pituitary‐related hormone levels, can cause clinical symptoms such as weight gain, fatigue, cold, constipation, facial edema, drowsiness, slow reaction, and mucinous edema. Unique clinical manifestations of puberty include sexual retardation and disorder, 6 cases of no secondary sexual characteristics in the prepuberty state,^7^, ^10^, ^15^, ^17^, ^21^ 3 cases without pubic hair and axillary hair growth,^9^, ^12^, ^13^ 1 case of penis not growing,^13^ and one case of pseudosexual precocious puberty.^12^ Special manifestations such as pericardial effusion, pleural effusion, acanthosis nigricans, hirsutism, Hoffmann syndrome, and VWGS have also been reported.^23^ Headache, vision loss, and menstrual disturbances are consistent with compression symptoms caused by gonadal axis disturbance and pituitary enlargement in PHPH. In conclusion, the clinical manifestations of PHPH in adolescent children include local compression symptoms caused by pituitary hyperplasia and related pituitary hormone levels besides the clinical symptoms of primary hypothyroidism. Sexual retardation and disorders and even related mental and psychological problems may also occur.

### 5.3 Investigations

In PHPH patients, FT3, FT4, T3, and T4 levels decreased while TSH increased. All 20 patients reviewed had decreased FT4 and/or T4 and increased TSH. Of these, 16 clearly reported positive anti‐thyroid antibodies, TPOAb and/or TGAb.^5^, ^6^, ^7^, ^8^, ^9^, ^11^, ^12^, ^13^, ^14^, ^15^, ^17^, ^18^, ^19^, ^20^, ^22^ PHPH is usually accompanied by elevated prolactin (PRL). For example, 14 cases in the review showed increased PRL,^6^, ^7^, ^9^, ^10^, ^12^, ^13^, ^15^, ^17^, ^18^, ^19^, ^20^, ^21^, ^22^ which can be misdiagnosed as prolactinoma leading to inappropriate treatment and irreparable loss. The pathogenesis of hyperprolactinemia in hypothyroidism is unclear. The mainstream hypothesis suggests that there are four types^24^: (1) TRH stimulates the secretion of prolactin cells; (2) dopamine synthesis disorder; (3) the clearance rate of prolactin is reduced; and (4) the inhibition of pituitary prolactin cell gene expression is weakened. Additionally, the increase of the pituitary gland presses the pituitary stalk, which affects the porosity circulation of the pituitary body and can also lead to a decrease in dopamine and an increase in prolactin.^25^

Meanwhile, GH, Insulin‐like growth factor 1 (IGF‐1), and IGF binding protein 3 (IGFBP3) levels decreased commonly. Five cases clearly reported GH deficiency,^7^, ^8^, ^10^, ^15^ and one case showed a decrease in IGF‐1.^13^ Thyroxine is one of the factors that stimulate GH synthesis. Therefore, hypothyroidism can obstruct GH synthesis and reduce pituitary GH secretion. Abnormalities in FSH, LH, and T are also been reported in prepubertal children and are mostly elevated.^1^, ^26^ However, in a study of 20 adolescent children, 7 showed decreased FSH,^5^, ^7^, ^10^, ^13^, ^14^, ^15^, ^17^ 6 had decreased LH,^5^, ^7^, ^10^, ^12^, ^13^, ^14^ and 2 had T deficiency.^12^, ^13^ These abnormalities were mainly associated with sexual developmental retardation and disorders. Two additional cases reported a deficiency of COR,^5^, ^18^ two cases with dyslipidemia,^9^, ^12^ decreased hemoglobin,^8^, ^12^ and one case with abnormal coagulation factors, liver function, and creatine phosphokinase (CPK).^8^, ^11^, ^12^ The patients reported herein had severe hypothyroidism, combined with increased PRL, decreased COR levels, and lower levels of LH, and normal GH levels.

In summary, increased prolactin is commonly seen in children with PHPH during both puberty and prepuberty. However, the difference is that pubertal children are more likely to have abnormal sex hormone levels, whereas prepubertal children are more likely to have combined growth hormone deficiency.

Diffuse enlargement of the anterior pituitary gland with mostly homogeneous masses was observed on intracranial MRI.^14^ The masses showed uniform enhancement with no signs of hemorrhage, necrosis, or cystic fibrosis. The saddle plate extension was visible, along with dome‐shaped blunt marginal changes.^15^ The posterior pituitary gland usually showed high signal. As the disease progresses, the pituitary stalk may be normal or thickened but not distorted. It may have a suprasellar extension to form a gourd‐like appearance and may extend to the suprasellar pool or occasionally compress the optic chiasm but rarely invade the cavernous sinus.^1^ Of the 20 cases reviewed, 10 had a pituitary mass extending to the suprasellar cistern and affecting the optic chiasm.^8^, ^10^, ^11^, ^13^, ^14^, ^15^, ^18^, ^19^, ^20^, ^21^ Six cases clearly reported lesions with uniform enhancement.^7^, ^11^, ^14^, ^17^, ^19^, ^21^ The patient's pituitary magnetic resonance imaging suggested solid space‐occupying lesions in and on the saddle with clear boundaries, equal T1 and T2 signals, enhanced signal, and a “dumbbell” shape. The lesion was slightly blocked and narrowed in the saddle compartment plane. The pituitary stalk and optic cross were compressed, and the saddle bottom was compressed and depressed.

### 5.4 Diagnosis and differential diagnosis

Diagnosing PHPH can be challenging. Clinical symptoms, laboratory test results, and imaging findings can provide a preliminary diagnosis. A key biochemical indicator is the hallmark of primary hypothyroidism: elevated TSH and low FT4/FT3 levels, which are not expected in pituitary adenoma. Symptoms of primary hypothyroidism include fatigue, weight gain, and cold intolerance. In contrast, pituitary hyperplasia, which occurs as a response to chronic TSH stimulation, can cause an enlarged pituitary gland with potential mass effects. This may lead to symptoms such as headaches, visual disturbances, and hormonal imbalances, including elevated prolactin levels. Confirmation of PHPH should be based on the response to thyroid hormone replacement therapy. If the patient's symptoms improve with appropriate treatment, and the pituitary gland is significantly reduced or even restored to normal, and related hormone levels return to normal, the diagnosis can be confirmed. When the diagnosis is uncertain, it is important to avoid making a hasty decision on surgical resection to prevent irreversible pituitary dysfunction, which can seriously affect the growth and development of adolescent children during this special period.

Pituitary gland enlargement is observed during puberty, pregnancy, and lactation due to physiological pituitary growth.^26^ It has been reported that the glands are significantly enlarged during puberty, up to 8 mm in males and 10 mm in females.^27^, ^28^ Pathological pituitary hyperplasia is usually seen in hypothyroidism, primary adrenal insufficiency, primary hypogonadism, prolonged use of exogenous estrogen, and pituitary congestion caused by traumatic, iatrogenic, or spontaneous cerebrospinal fluid leaks.^27^ Patients with TSH‐secreting pituitary adenomas may have elevated TSH levels (usually less than 50 mIU/mL) and elevated levels of FT3, FT4, T3, and T4.^28^ Other hormones such as plasma PRL, GH or FSH and LH may be elevated in patients with pituitary prolactinomas, growth hormone‐secreting pituitary adenomas or gonadotropin‐secreting adenomas respectively. The pituitary mass as a space‐occupying lesion results in decreased pituitary function and impaired pituitary TSH secretion followed by a decrease in the plasma levels of FT3, FT4, T3, and T4. Distinguishing PHPH from pituitary adenomas poses challenges due to overlapping clinical and laboratory features. Therefore, the response to thyroid hormone replacement therapy plays a crucial role. Improvement following treatment supports the diagnosis of PHPH, whereas inadequate response may suggest a pituitary adenoma.^29^

### 5.5 Treatment and outcome

Thyroxine replacement therapy is the primary treatment for PHPH in adolescence. Starting with low doses, TSH, FT4, PRL, FSH, LH, T, and other pituitary hormones are monitored along with pituitary MR and pubertal growth and development changes. Among the 20 studies reviewed, the pituitary glands of 16 patients returned to normal size after follow‐up.^5^, ^6^, ^7^, ^8^, ^9^, ^11^, ^14^, ^15^, ^16^, ^17^, ^19^, ^20^, ^21^, ^22^, ^30^ The minimum recovery time was 1 month and the maximum was 3 years,^7^, ^21^ with most patients recovering between 3 and 6 months.^5^, ^6^, ^7^, ^8^, ^9^, ^11^, ^14^, ^17^, ^19^, ^22^

In one case study, men with adolescent growth retardation normalized to FT4, TSH, FSH, LH, T, and IGF‐1.^13^ After 16 months of treatment, their height increased by 13 cm, the volume of bilateral testis increased by about 5 mL, extended penis increased by 5 cm, and pubic hair began to grow on the external genitalia from stage Tanner I to stage Tanner IV. This confirmed that the growth and development of PHPH can be achieved after levothyroxine replacement. The other four children also showed catch‐up growth in height after the treatment.^11^, ^12^, ^13^, ^16^, ^17^, ^22^

Adolescents face complex psychological changes, including self‐awareness, sensitivity, suspicion, and physiological problems caused by the disease. They may also experience low self‐esteem, anxiety, depression, serious mental illness, and suicidal tendencies.^31^ Therefore, we believe that during the treatment process, it is important to communicate with children and their families and seek psychological counseling from experts if necessary. This can help enhance the confidence of overcoming the disease and reduce the occurrence of tragedy.

After ensuring optimal thyroxine substitution, if serum TSH level has only partially decreased with no significant reduction in the enlarged pituitary gland, one should consider the possibility of PHPH combined with pituitary TSH‐secreting adenoma.^1^ If TSH levels continue to rise and the enlarged pituitary gland continues to grow, pituitary TSH adenoma should be considered, and surgical treatment is recommended in these cases.^32^

In conclusion, we have summarized the pathogenesis, clinical manifestations, laboratory tests, imaging findings, diagnosis and differential diagnosis, and therapeutic follow‐up of PHPH in adolescents. This summary may help identify PHPH in adolescents, guide treatment decisions, and minimize the adverse consequences of misdiagnosis. Although PHPH is not common in adolescent children, further reliable evidence is needed to accurately understand disease progression and establish scientific diagnostic criteria. While these symptoms are reversible after thyroid hormone replacement therapy, some adolescents with severe dysplasia or short stature may still experience difficulty with normal development and growth. Therefore, further studies are urgently needed to improve the necessity and timing of early diagnosis of PHPH and associated hormone deficiency in adolescent children.

## AUTHOR CONTRIBUTIONS

**Xiangfeng Yuan:** Data curation; writing – original draft; writing – review and editing. **Jiangyu Zhu:** Data curation; writing – original draft; writing – review and editing. **Xiaoyu Su:** Data curation; writing – original draft; writing – review and editing. **Huiling Tan:** Writing – review and editing. **Siqi Wang:** Data curation; writing – original draft. **Xueying Zheng:** Data curation; writing – original draft. **Yu Ding:** Conceptualization; methodology. **Sumei Li:** Conceptualization; methodology.

## FUNDING INFORMATION

The authors received no specific funding for this work.

## CONFLICT OF INTEREST STATEMENT

The authors declare that they have no competing interests.

## ETHICS STATEMENT

Institutional Review Board (IRB) at the First Affiliated Hospital of USTC approved the study for the retrospective review (2023‐RE‐382).

## CONSENT

Written informed consent was obtained from the patient to publish this report in accordance with the journal's patient consent policy.

## Data Availability

We have presented all the data in the text.

## APPENDIX A A comprehensive review of 20 cases of adolescent PHPH characteristic

### A.1

**Table:**

Order number	Author, year	Age (year)	Gender	Sings and symptoms	Pubertal development status	Imaging examination	Laboratory examination	Treatment	Treatment effect
1	Farley, 1998^15^	12	Male	Fear of cold, mild anemia, pale and dry skin, visible carrot color	Height: 120.4 cm, Bone age delay, Prepubertal state	CT: the pituitary gland was enlarged and extended to the saddle Other: Perimetry suggested a superior visual field defect	FT4↓, TSH↑, TGAb+, TMAb+, PRL↑, GH↓, FSH↓	Oral levothyroxine	After 6 weeks, the pituitary size returned to normal;FT4 returned to normal, TSH was close to normal; upper visual field defect improved and clinical symptoms were significantly relieved
2	Bhansali, 2004^5^	13	Female	Weight gain, drowsiness, constipation, fear of cold, rough and dry skin	Height: 140 cm (the average height of parents is 158 cm), Bone age delay of 2 years, Adolescent, The pubertal state is B2A1P2 (stage TannerII)	Imaging showed: sellar mass, size of (6 × 17 × 9 mm), considered for non‐function pituitary tumor	FT4↓, TSH↑, TMAb+	Oral levothyroxine	After 6 months, the pituitary size returned to normal; FT4 and TSH returned to normal
3	Bhansali, 2004^5^	13	Male	Weight gain, drowsiness, fatigue, snoring, constipation, hoarseness	Height: 145 cm (the average height of parents is 168 cm), Bone age is normal, The pubertal status is A1P2 The bilateral testicular volume is 4 mL	MRI: significant pituitary enlargement, Size is (14.1 × 18.5 × 10.1 mm), extending to the saddle	FT4↓, TSH↑, TMAb+, FSH↓, LH↓, COR (8 AM)↓	Oral levothyroxine	After 6 months, the pituitary size returned to normal; FT4 and TSH returned to normal
4	Ashley, 2005^21^	12	Female	Headache, fatigue	Height ominous, No secondary sexual characteristics	MRI: (1) showing uniform enhancement of the saddle mass, extending to the saddle, pressing the optic cross, and showing a pointed upper edge (2) There was no expansion or erosion of the butterfly saddle (3) Did not see saddle side involvement	FT4↓, TSH↑, PRL↑	Oral levothyroxine	After 1 month, the pituitary size returned to normal; clinical symptoms resolved
5	Jia, 2007^9^	16	Female	Weight gain, dizziness, slow mobility, hypomnesis, sluggish responses, constipation, rough skin	Height: 155 cm; Menstruation regularity, menstrual period is extended for a few days than before; No pubic hair, axillary hair	MRI: the butterfly saddle was small, the pituitary volume increased but symmetrical, the height was about 14 mm, and the pituitary stalk was centered Others: B‐ultrasound suggests a small amount of pericardial effusion	FT4↓, TSH↑, TGAb+, TMAb+, TRAb‐, TSI−, PRL↑, TC↑, TG↑, LDL‐C↑	Oral levothyroxine	After 3 months, the pituitary size returned to normal; FT4, TSH and PRL returned to normal; and the clinical symptoms were significantly relieved
6	Lee, 2008^16^	10.3	Female	Bitemporal hemianopsia	Height: 121 cm, Bone age was 7 years and 10 months, Bilateral breast enlargement (15 cm × 15 cm, stage Tanner II), pubic hair (stage Tanner I), and no precocious puberty appeared in the external genitals, The sign of the uterus was (2.62 × 0.96) cm	MRI revealed a mass in the (3.8 × 9.6 × 12.1 mm)	FT4↓, TSH↑, TPOAb−, TGAb−	Oral levothyroxine	After 2 months, FT4 and TSH returned to normal;The growth rate was increased to 0.8 cm/month, Bilateral breast enlargement (25 cm × 25 cm, Tanner II), pubic hair (Tanner I); After 6 months, the pituitary size was normalized and the growth rate was normalized
7	Simsek, 2009^7^	14.5	Male	Pale and swollen face, mild headaches, fatigue, fear of cold, chronic constipation	Short stature (height: 135 cm), Adolescence was delayed, No secondary sexual signs appeared	MRI: the pituitary mass was large and homogeneous, and pituitary adenoma was considered	FT4↓, TSH↑, PRL, GH, FSH, and LH deficiency	Oral levothyroxine	After 6 months, pituitary enlargement disappeared; After 3 years, clinical symptoms disappeared; FT4, TSH and PRL normalized
8	Simsek, 2009^7^	13	Female	Excessive menstruation, fear of cold, fatigue	Short stature (height: 146 cm), Breast (Tanner IV phase), Pubis (Tanner III phase)	MRI: enlarged pituitary enlargement (11 mm) to the saddle on the extension	FT4↓, TSH↑, TPOAb+, PRL↑, GH↓	Oral levothyroxine	After 3 years, pituitary enlargement disappeared; menstruation to normal, clinical symptoms disappeared; FT4, TSH and PRL returned to normal
9	Babu, 2010^19^	15	Female	Headaches, weight gain, blurred vision, dry and rough skin, mild hirsutism	Height ominous, Menstrual disorder	MRI:a mass in the sellar region, extending to the saddle, with uniform enhancement, suggesting a pituitary large adenoma Others: Visual field tests suggest a mildly reversible anterior visual access pathway oppress	FT4↓, TSH↑, TPOAb+, PRL↑	Oral levothyroxine	After 3 weeks, the PRL returned to normal; After 3 months, the pituitary size returned to normal; FT4 and TSH returned to normal and clinical symptoms were significantly relieved; the visual field and eye movements were normal
10	Wang, 2010^17^	13	Female	growth retardation, fear of cold	Height: 129 cm; Prepubertal state	MRI: saddle area occupancy, T 1 WI and Other signals, T 2 WI slightly higher signal, pituitary uniform strengthening	FT4↓, TSH↑, TGAb+, PRL↑, FSH↓	Oral levothyroxine	After 6 months, the pituitary size returned to normal; FT4, TSH and PRL normalized; height increased by 5 cm
11	Cekmez, 2011^10^	12	Female	growth retardation, dry hair	Height: 129.4 cm; Bone age delay of 4 years; Prepubertal state	MRI: an anterior pituitary mass of size (16 × 12 mm) with the lesion slightly extending to the right supraselllar pool	FT4↓, TSH↑, TPOAb‐, PRL, GH, LH and FSH were lower	Oral levothyroxine	After 3 months, the pituitary mass was significantly reduced;FT4 and TSH returned to normal
12	Shen, 2013^22^	12	Male	fatigue, anorexia, asthma after activity, yellow skin, intermittent eyelid edema	Height ominous	MRI shows: pituitary abnormal strengthening nodule, size is (1.7 × 1.6 × 1.6 cm) Other: CT suggested pericardial effusion and bilateral pleural effusion	FT4↓, TSH↑, TPOAb+, TGAb+, PRL↑	Oral levothyroxine	After 2 months, the pituitary mass shrank significantly, FT4 and TSH returned to normal, and the clinical symptoms disappeared; After 3 months, the size and shape of the pituitary gland returned to normal; One year later, the height increased by 8 cm higher than before
13	Cebeci, 2013^11^	12	Male	Headache, blurred vision, bloating, fatigue, Hoffman syndrome, pale face, dry skin, bilateral pseudoenlarged shoulder and calf muscles, proximal muscle weakness	Height: 140 cm, Bone age delay of 2 years, Pubertal status (stage Tanner III)	MRI: the pituitary gland was enlarged, 12 mm, extending to the suprasellar pool, and had no obvious compression It is even enhanced Other: ophthalmological examination suggested bilateral optic papilledema	FT4↓, TSH↑, anti‐thyroid antibody+	Oral levothyroxine and acetazolamide	After 3 months, the clinical symptoms had disappeared; After 6 months, the pituitary size (5 mm) was normalized, the growth rate was 6 cm/year, and the CPK levels were decreased
14	Sterkenburg, 2014^14^	13	Female	Headache, fatigue	Short stature (Height ominous)	MRI: a homogeneous mass in the selellar pituitary, enlarging upward and almost reaching the optic chiasm	FT4↓, TSH↑, TPOAb+, LH and FSH were at the prepubertal level	Oral levothyroxine	After 3 months, the pituitary size returned to normal
15	Zhang, 2017^12^	14	Male	Gain weight, growth retardation, constipation, muscle weakness, poor academic performance, and VWG syndrome	Height: 139 cm, Bone age delay of 4 years, Pseudoprecocious puberty, with a bilateral testicular volume of 25 mL and a stretched penile length of 5 cm, with no pubic hair or armpit hair	MRI: pituitary enlargement, and pituitary hyperplasia was considered Other: B‐ultrasound suggested hepatomegaly	FT4↓, TSH↑, TPOAb+, TGAb+, PRL↑, LH↓, T↓, TC↑, AST↑, ALT↑, HGB↓	Oral levothyroxine	After 6 months, the pituitary mass was significantly reduced;FT4 and TSH returned to normal; height increased by 5 cm
16	Bhattacharya, 2020^6^	11	Female	Drowsiness, poor learning, fear of cold, weight gain, full moon face, hoarseness	Height: 128 cm, Menstrual rule	MRI: a sellar mass, the size of (1.58 × 1.43 × 3.11 cm), a small area of necrosis, and contrast enhancement	T4↓, TSH↑, TPOAb+, PRL↑	Oral levothyroxine	After 2 months, T4 was normal; After 6 months, the pituitary size returned to normal; TSH and PRL returned to normal
17	Yu, 2020^13^	15	Male	Obesity, poor appetite, hypomnesis, insomnia	Short stature (height: 148 cm), The penis does not grow (the stretched penis is 25 cm in length), No pubic hair in the external genital organs (stage Tanner I), The left testicular volume is 10 mL and right testicular volume is 9 mL	MRI: pituitary fossa mass, T1 signal in sella, T2 signal is slightly longer, uneven and slightly strengthened; the pituitary stalk is not clear and the optic cross moves up	FT4↓, TSH↑, TPOAb+, TGAb+, FSH↓, LH↓, T↓, IGF−1↓, PRL↑	Oral levothyroxine	After 6 months, the clinical symptoms were significantly relieved, the pituitary gland was significantly decreased, and the levels of FT4, TSH, FSH, LH, T, and IGF‐1 returned to normal; After 16 months, the height increased to 161 cm, and the penis grew (the extended penis was 7 cm long), Pubic hair begins to grow on the external genitalia (Tanner IV Phase 1);The volume of the bilateral testis was 15 mL
18	Barbero, 2022^8^	13	Female	Hypermenorrhea, xerosis cutis, acanthosis nigricans, thin hair	Height: 133.8 cm (average height of parents: 155 cm), Bone age was 96 years old, Menarche, 17 years ago	MRI: the gland in contact with the optic cross was enlarged with a height of 16 mm Other: B ultrasound indicates that the size of the right ovarian cyst is (40 × 28 mm)	FT4↓, TSH↑, TPOAb+, TGAb+, GH↓, HGB↓, APTT↑, FIB↓, vWF ↓	Oral levothyroxine	After 1 month, FT4, APTT, FIB, and vWF returned to normal; TSH returned to normal after 7 weeks; The pituitary size was normalized after 3 months
19	Ms, 2022^18^	17	Female	Headache, bitemporal hemianopia, fatigue, face swelling, weight gain, dry and rough skin, hoarseness, alopecia, mucinous oedema	Height ominous, Menstrual disorder	MRI: a diffuse enhancing lesion in the sellar region extending into the optic chiasm	FT4↓, TSH↑, TPOAb +, PRL, Morning COR↓	Oral levothyroxine and hydrocortisone	After 7 months, the pituitary mass was significantly reduced; regular menstruation, normal vision, and clinical symptoms were significantly relieved
20	Wang, 2022^20^	17	Female	Headache, anemia, pale face, swollen face	Height ominous, menstrual unknown	MRI: the soft tissue mass was seen in the saddle, the size of (0.8 × 1.8 × 1.4 cm) was significantly strengthened, the upward growth of the saddle diaphragm, the pituitary stalk was shortened and centered, no obvious subsidence at the bottom of the saddle, and the mass compressed the optic cross	FT4↓, TSH↑, TPOAb+, TGAb+, PRL↑	Oral levothyroxine	After 9 months, FT4 returned to normal and TSH decreased significantly; clinical symptoms resolved significantly After 2 years, the pituitary size returned to normal

Abbreviations: ALT, alanine aminotransferase; APTT, activated partial thromboplastin time; AST, aspartate transaminase; COR, cortisol; FIB, fibrinogen; FSH, follicle stimulating hormone; FT4, free thyroxine; GH, growth hormone; HGB, hemoglobin; LDL‐C, low‐density lipoprotein cholesterol; LH, luteinizing hormone, PRL, prolactin; T, testosterone; TC, total cholesterol; TG, triglyceride; TGAb, thyroglobulin antibody; TMAb, thyroid microsomal antibodies; TPOAb, thyroid peroxide antibody; TRAb, thyrotropin receptor antibody; TSH, thyroid‐stimulating hormone; TSI, thyroid stimulating immunoglobulin; VWF, von Willebrand factor.
